# Lym-1 Chimeric Antigen Receptor T Cells Exhibit Potent Anti-Tumor Effects against B-Cell Lymphoma

**DOI:** 10.3390/ijms18122773

**Published:** 2017-12-20

**Authors:** Long Zheng, Peisheng Hu, Brandon Wolfe, Caryn Gonsalves, Luqing Ren, Leslie A. Khawli, Harvey R. Kaslow, Alan L. Epstein

**Affiliations:** 1 Department of Pathology, Keck School of Medicine, University of Southern California, Los Angeles, CA 90033, USA; longzhen@usc.edu (L.Z.); Peisheng@usc.edu (P.H.); bwolfe@usc.edu (B.W.); gonsalves.caryn@gmail.com (C.G.); renluqing@hotmail.com (L.R.); 2 Department of Physiology and Neuroscience, Keck School of Medicine, University of Southern California, Los Angeles, CA 90033, USA; hrkaslow@usc.edu

**Keywords:** cancer immunotherapy, lymphoma, CAR T cells, Lym-1

## Abstract

T cells expressing chimeric antigen receptors (CARs) recognizing CD19 epitopes have produced remarkable anti-tumor effects in patients with B-cell malignancies. However, cancer cells lacking recognized epitopes can emerge, leading to relapse and death. Thus, CAR T cells targeting different epitopes on different antigens could improve immunotherapy. The Lym-1 antibody targets a conformational epitope of Human Leukocyte Antigen-antigen D Related (HLA-DR) on the surface of human B-cell lymphomas. Lym-1 CAR T cells were thus generated for evaluation of cytotoxic activity towards lymphoma cells in vitro and in vivo. Human T cells from healthy donors were transduced to express a Lym-1 CAR, and assessed for epitope-driven function in culture and towards Raji xenografts in NOD-scidIL2Rgamma^null^ (NSG) mice. Lym-1 CAR T cells exhibited epitope-driven activation and lytic function against human B-cell lymphoma cell lines in culture and mediated complete regression of Raji/Luciferase-Green fluorescent protein (Raji/Luc-GFP) in NSG mice with similar or better reactivity than CD19 CAR T cells. Lym-1 CAR transduction of T cells is a promising immunotherapy for patients with Lym-1 epitope positive B-cell malignancies.

## 1. Introduction

B-cell non-Hodgkin lymphomas (B-NHL) encompass a heterogeneous group of cancers with increasing incidence [1,2]. R-CHOP (Rituximab, cyclophosphamide, Adriamycin, vincristine, and prednisone) is the frontline treatment regimen with a high initial response rate [3]. However, 30–60% of responding patients relapse, and are refractory to subsequent R-CHOP treatment [3]. Patients may be eligible for allogenic stem cell transplantation (ASCT), but the prognosis is dismal, with 9.9 months median overall survival (OS) [4]. Thus, new modalities are needed to address this unmet medical need.

Chimeric antigen receptors (CARs) are synthetic molecules containing three distinct modules: an extracellular antibody-based recognition site, a transmembrane module that anchors the molecule into the cell membrane, and a chimeric intracellular signaling domain that transmits the activation signal [5]. Upon engagement of an epitope on a tumor cell, CAR-directed T cells can induce tumor cell apoptosis or death through cytotoxic T-cell machinery, in a MHC-independent manner [6]. In the past few years, CAR T cells targeting Cluster of Differentiation 19 (CD19) have achieved impressive outcomes in the treatment of patients with relapsed or refractory (R/R) acute lymphoblastic leukemia (ALL) [7,8,9]. Although CD19 CAR T cell treatment of R/R B-NHL has been less successful, it has improved objective response rates (ORR) from 20–30% [10] to 79%, with complete remission rates of 30–50% [11,12,13,14] which is 7-fold higher than historical results [10,11]. The successful treatment of B cell malignancies with CD19 CAR T-cell therapy provides direct evidence for the effectiveness of this strategy.

Lym-1, a murine IgG2a monoclonal antibody, was generated by immunizing mice with nuclei isolated from Raji lymphoma cells [15]. Lym-1 binds to a discontinuous conformational epitope on several HLA-DR subtypes [16] with a greater binding affinity for malignant B cells than normal B cells [16]. In clinical studies, 80% of patients with non-Hodgkin lymphoma (NHL) and 40% of patients with chronic lymphocytic leukemia (CLL) were found to be Lym-1 positive [17]. A clinical trial performed several decades ago using murine Lym-1 resulted in limited therapeutic effects in 10 patients with refractory B-cell lymphoma, perhaps due to the short half-life and immunogenicity of the murine antibody [18]. Subsequently, a chimeric version of Lym-1 was tested clinically after labeling with ^131^I [19] or ^67^Cu-2IT-BAT [20] to treat resistant intermediate and high-grade lymphoma, where it was found to achieve significant efficacy in the treatment of chemotherapy-resistant tumors. This record makes Lym-1 an attractive antibody to test using CAR T-cell technology.

This paper reports the design and evaluation of a second generation Lym-1 CAR using human primary T cells. The results demonstrate that Lym-1 CAR T cells display epitope-driven cytokine release, proliferation, and cytotoxicity against Lym-1 epitope-positive cell lines, and complete eradication of Lym-1 epitope-positive Raji xenograft tumors in NSG mice.

## 2. Results

### 2.1. Efficient Expression of Lym-1 CAR on Transduced Primary Human T Cells

**Figure 1:**
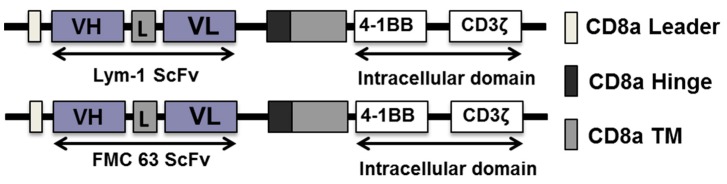
Schematic representation of Lym-1 CAR and CD19 (FMC 63) CAR constructs.

The Lym-1 CAR construct used in this study was a second-generation construct with a CD8α leader sequence, a Lym-1 specific scFv moiety fused to a CD8α hinge and transmembrane domain, followed by a 4-1BB co-stimulation and CD3ζ signaling domains (Figure 1).

**Figure 2:**
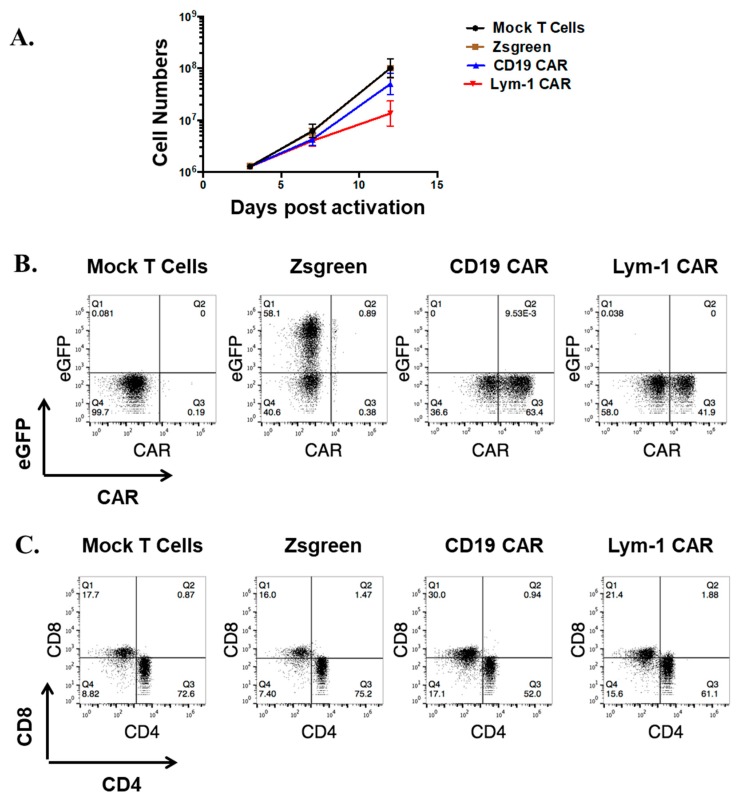
Efficient Lym-1 CAR expression on human primary T cells. (**A**) Cell numbers were recorded after mock or virus transduction. (*n* = 3 replicates per point; representative of three donors); (**B**) At day 10, 10^6^ T cells were labeled with 2 μg biotin-protein L, followed by detection with Allophycocyanin (APC)-streptavidin. Mock-transduced T cells served as a negative control. (*n* = 6); (**C**) After expansion, the CD4/CD8 ratio of the T-cell preparations shown in Panel B were analyzed for CD4 and CD8 expression (representative of three donors).

To transduce T cells to express the CAR, the CAR cDNA was inserted into a modified self-inactivating pLVX lentiviral vector, between the EcoRI and MluI sites. The replication-incompetent lentiviruses encoding the CARs or pLVX-EF1α-IRES-ZsGreen (as a control) were used to transduce human primary T cells three days after activation (day 0). Compared to mock transduced T cells (T cells processed without addition of a lentiviral transfer vector), Lym-1 CAR and CD19 CAR transduced T cells showed similar rates of proliferation up to day 7. After day 7, CD19 CAR T cells proliferated more rapidly than Lym-1 CAR T cells (Figure 2A). Lym-1 and CD19 CAR T cells showed consistent transduction efficiencies ranging from 30% to 80%, as determined by flow cytometry using protein L (Figure 2B). At day 12, the T-cell preparations consisted of a mixture of CD4+ (~65%) and CD8+ (~20%) T cells (Figure 2C).

### 2.2. Epitope-Driven Upregulation of CD107a and Epitope-Dependent Cytotoxicity of Lym-1 and CD19 CAR T Cells

**Figure 3:**
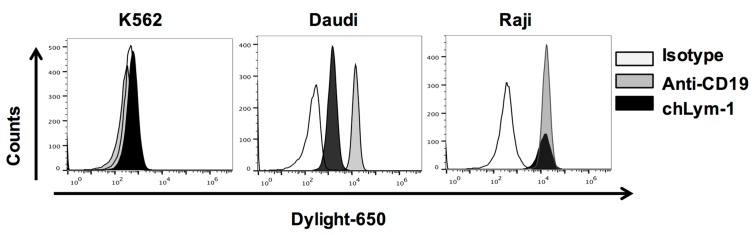
Detection of Lym-1 and CD19 epitopes on Daudi and Raji cells, but not K562 cells. Cell surface epitope intensity was detected by incubation with Dylight 650 conjugated chLym-1 antibody or APC conjugated anti-CD19 antibody.

**Figure 4:**
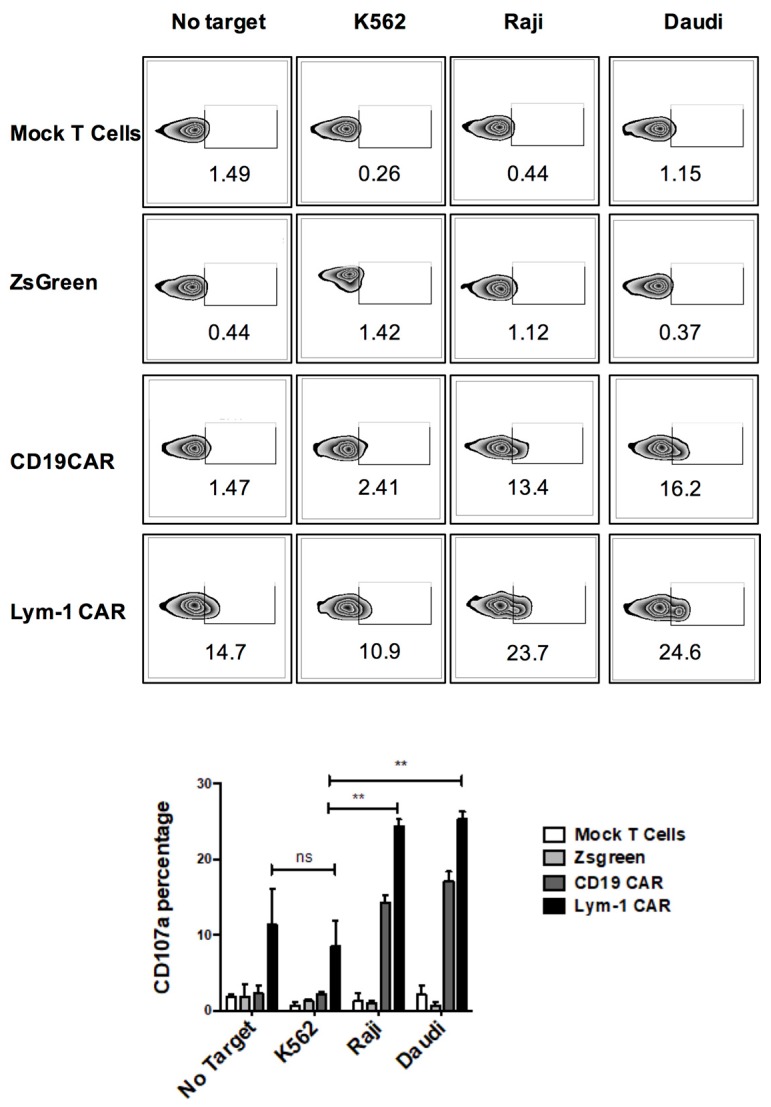
Epitope-driven upregulation of CD107a on Lym-1 and CD19 CAR T cells. Lym-1 and CD19 CAR T cells were detected by protein L and APC-streptavidin flow cytometry. Mock transduced T cells were added to each preparation to adjust the CAR T cell fraction to 30%. T cells (2 × 10^5^) were then incubated with 10^5^ Raji or Daudi cells. Mock transduced T cells alone and CAR transduced T cells incubated with epitope-negative K562 cells served as negative controls. An anti-CD107a antibody and monensin were then added to the wells soon after. After a 5 h incubation, cells were labeled with PE-anti-CD3 antibody to differentiate tumor and T cells using flow cytometry. (**Top panel**) examples of data; (**Bottom panel**): data from *n* = 3 (ns, not significant; ** = *p* < 0.01; compared to CD107a level when co-incubated with K562).

**Figure 5:**
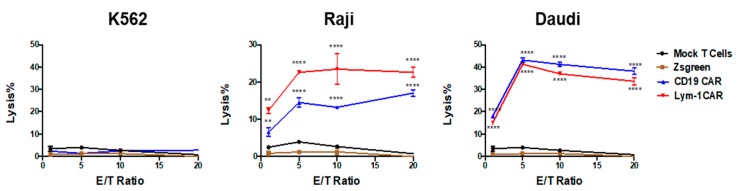
Epitope-driven cytotoxicity of Lym-1 and CD19 CAR T cells. T cells (control or 30% CAR positive) were cultured overnight with 2 × 10^4^ K562, Raji, or Daudi cells at indicated ratio. Supernatants were processed to measure cytotoxicity. Data from one donor is shown; similar results were obtained from a second donor. For each donor, three independent transductions were each assessed using triplicate determinations. ** = *p* < 0.01; **** = *p* < 0.001 compared to % lysis in mock-transduced T cells at the same E/T ratio.

Three cell lines were used to assess epitope-driven functions of Lym-1 and CD19 CAR T cells. Flow cytometry using chLym-1 and anti-CD19 antibodies identified two cell lines expressing Lym-1 and CD19 epitopes, Raji and Daudi, and one that expressed neither, K562 (Figure 3). pLVX-EF1α-IRES-ZsGreen transduced T cells and mock transduced T cells were used to detect T-cell activity independent of either the Lym-1 or CD19 CAR. Both Lym-1 and CD19 CAR T cells significantly up-regulated CD107a in response to co-culture with Raji and Daudi (*p* < 0.01) but not K562 (Figure 4). Similarly, the Lym-1 and CD19 CAR T cells efficiently lysed the epitope-expressing Raji and Daudi cell lines, but not the epitope-negative K562 cell line. Mock transduced T cells and pLVX-EF1α-IRES-ZsGreen transduced T cells did not show a significant level of cell lysis at any of the target/effector cell ratios tested (Figure 5).

### 2.3. Epitope-Driven Release of Cytokines from Lym-1 and CD19 CAR T Cells

**Figure 6:**
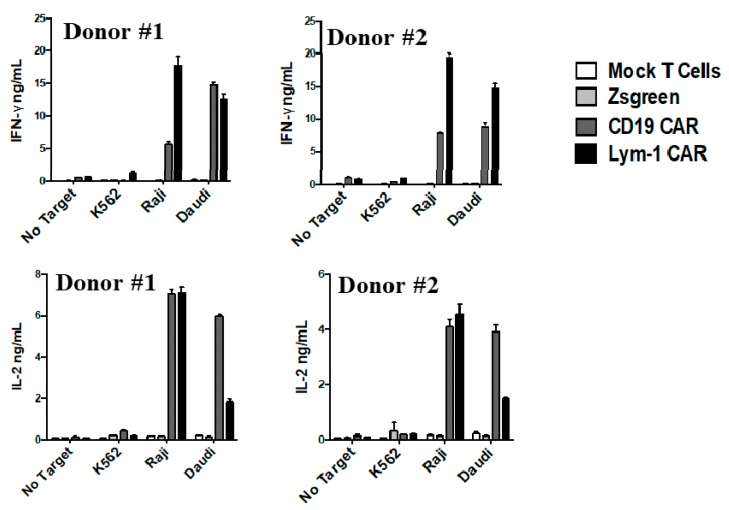
Epitope-driven release of cytokines from Lym-1 and CD19 CAR T cells. The percentage of CAR-transduced T cells was adjusted to 30%. Cells (2 × 10^5^) were then incubated with 10^5^ K562, Raji, Daudi, or no target cells. Representative cytokine release levels from two donors are shown.

Lym-1 and CD19 CAR T cells were incubated with tumor cell lines at a ratio of 2:1, as described above. T cell preparations comprised of either Lym-1 CAR (30% CAR positive) or CD19 CAR (30% CAR positive). T cells released IFN-γ and IL-2 when co-cultured overnight with epitope-positive Raji and Daudi cells, but not with K562 or in the absence of a target tumor cell line. Neither Zsgreen or mock-transduced T cells released IFN-γ or IL-2 when cultured with any of the three tumor cell lines (Figure 6). Therefore, release of these cytokines by Lym-1 and CD19 CAR T cells appears to be due to recognition of a Lym-1 or CD19 epitope.

### 2.4. Epitope-Driven Proliferation of Lym-1 and CD19 CAR T Cells

**Figure 7:**
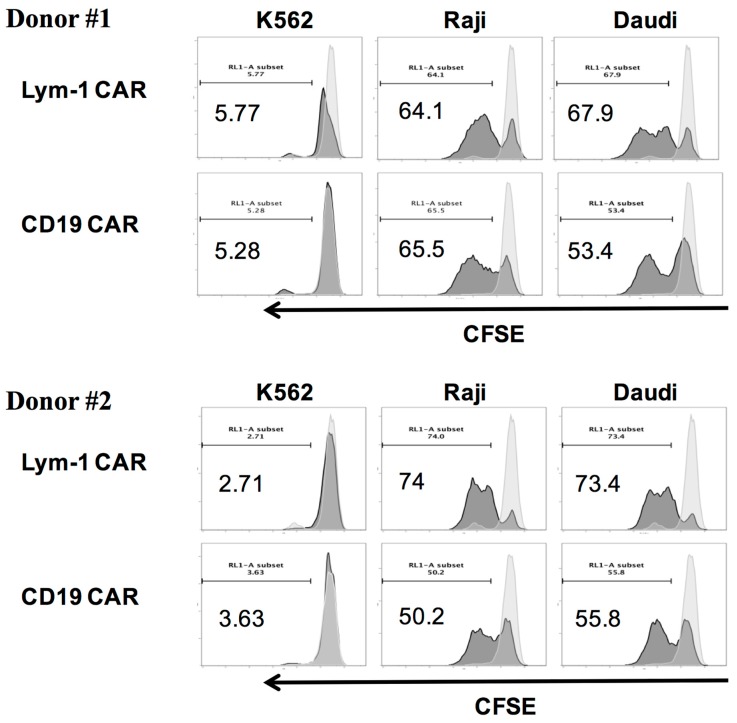
Epitope-driven proliferation of Lym-1 and CD19 CAR T cells. Lym-1 or CD19 CAR T cells (10^6^) were labeled with CSFE-Far-red dye and co-cultured with 10^6^ irradiated K562, Daudi, or Raji cells in the absence of exogenous cytokines. CAR T cells cultured alone served as a negative control. After five days of co-culture, cells were labeled with PE-anti CD3 and analyzed by flow cytometry.

Lym-1 CAR and CD19 CAR T cells labeled with CFSE-Far-red cell proliferation trace dye were cultured alone, or with irradiated tumor cell lines with no cytokines added. After 5 days, the CFSE signal was measured, and overlap histograms prepared to represent the fluorescence intensity of CAR T cells alone compared to CAR T cells with indicated target T cells after five days of co-culture. When co-cultured with Raji and Daudi cells, Lym-1 and CD19 CAR T cells showed a significant shift to the left, indicating cell division and proliferation. However, little or no shift was observed when the CAR T cells were co-cultured with epitope negative K562 cells (Figure 7). These findings demonstrate Lym-1 and CD19 CAR T cells proliferate when cultured with cells expressing a recognized epitope.

### 2.5. Lym-1 and CD19 CAR T Cells Eradicate Raji/Luc-GFP Xenograft Tumors in NSG Mice

**Figure 8:**
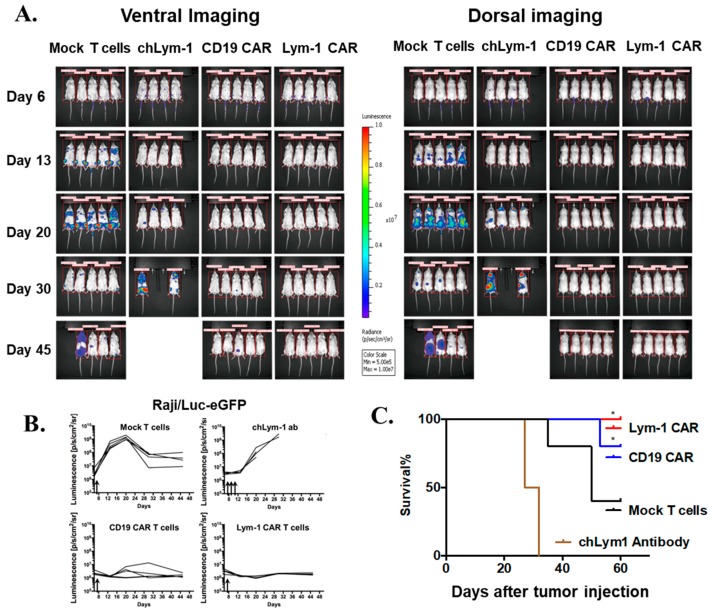
Lym-1 and CD19 CAR T cells eradicate Raji/Luc-GFP xenograft tumors in vivo. (**A**) Ventral and dorsal bioluminescence imaging of tumor burden in control and treated mice. Raji/Luc-GFP cells (10^6^) were injected intravenously into 8–10 week old male NSG mice (day 0). Luciferase bioluminescence was measured at day 6 to assess pre-treatment tumor burden. On day 7, 10^7^ mock-transduced T cells, CD19 CAR T cells, or Lym-1 CAR T cells in 100 μL phosphate buffered saline (PBS) were injected intravenously (*n* = 5)*.* For the chLym-1 antibody group, 100 μg chLym-1 was injected intravenously in 100 μl PBS on days 7, 9, and 11. (**B**) Quantification of bioluminescence shown in (**A**). (**C**) Kaplan–Meier plot of survival of mice. * = *p* < 0.01, compared to mock T cells group.

The anti-tumor effects of CAR T cells were evaluated using an NSG mouse leukemic Raji/Luc-GFP xenograft model. Luciferase activity of Raji/Luc-GFP was measured and titered in vitro, to confirm the feasibility of in vivo optical imaging. Tumor burden was assessed by serial bioluminescent imaging of luciferase expression six days after tumor inoculation. Equal tumor burden was verified in each group before treatment as evidenced by the luciferase signal (Figure 8A). On day 7, a single dose of 10^7^ mock-transduced T cells and Lym-1 CAR and CD19 CAR T cells (50% CAR positive cells as measured by Protein L) were injected intravenously (iv).

When the Lym-1 antibody binds to a Lym-1 epitope, it can induce target cell apoptosis or autophagy in addition to ADCC or CDC effects [21,22]. To determine if this effect of Lym-1 was sufficient to eradicate Raji cells in this in vivo model, a group of mice received 100 µg human chimeric Lym-1 (chLym-1) on days 7, 9, and 11.

In mice treated with Lym-1 CAR T cells, only background bioluminescence was detected, and the antitumor effect was durable throughout the 60 day experiment (Figure 8A–C). Essentially equal tumor eradication was achieved in the CD19 CAR T cell group, except that one mouse relapsed 30 days after treatment and died on day 53 (Figure 8C). In contrast, treatment with the chLym-1 antibody controlled tumor progression only up to day 13, whereupon tumor growth began (Figure 8A,B). These mice were sacrificed on days 25 and 30, due to paralysis of the hind legs and severe systemic tumor burden. In the five mice receiving mock-transduced T cells, tumor burden progressed until day 20, and then modestly regressed, with tumor resurgence in three mice causing death (Figure 8A,C). This result was likely due to an allogeneic reaction of the human T cells to Raji cells [23].

## 3. Discussion

Lym-1 is a mouse IgG2a antibody developed in the early 1980s [15]. Clinical studies confirmed its safety and potential benefit in the treatment of B-cell malignancies [24]. In addition, when bound to polymorphic variants of HLA-DR subtypes, the Lym-1 antibody has not been found to cause antigen shedding or modulation [18], important characteristics for a CAR T cell therapy. Therefore, a second generation Lym-1 CAR was designed for evaluation as a potential therapeutic for Lym-1 epitope positive B-cell malignancies.

This report demonstrates a Lym-1 CAR construct can be efficiently expressed in human primary T cells from healthy donors with transduction efficiencies ranging from 30–80%. The CD8/CD4 ratio of the preparation was approximately 2:5 after expansion (Figure 2B,C). During expansion, Lym-1 CAR T cells proliferated at a lower rate than mock-transduced T cells (Figure 2A). Since the Lym-1 epitope was not detected on the T cells from the three different donors used in this study, the reduced proliferation rate may arise from tonic signaling from chimeric antigen receptors induced by the clustering of scFv [25]. 4-1BB, as a co-stimulation domain, was reported to enhance persistence of engineered T cells in vivo [26], and resulted in an improved survival rate in CD19 CAR clinical studies [8]. In addition, 4-1BB signal activation can ameliorate T-cell exhaustion, induced by the tonic signaling of chimeric antigen receptors, which is a common phenomenon in most of the CARs products, except the highly functional CD19 CAR [25]. Therefore, a 4-1BB co-stimulation domain was chosen for this Lym-1 CAR construct.

The modest increase in CD107a expression on Lym-1 transduced T cells (10% v.s. 3%) before encountering the Lym-1 epitope positive tumor cell lines (Figure 4) may also arise from tonic signaling. Nonetheless, this increase did not affect epitope-driven activity of Lym-1 CAR T cells as monitored by secretion of IFN-γ and IL-2 (Figure 6), or lysis of Lym-1 epitope negative K562 cells (Figure 5). Importantly, the observed lower levels of expansion of Lym-1 CAR T cells during manufacture did not reduce proliferation upon recognition of the Lym-1 epitope: Lym-1 and CD19 CAR T cells proliferated at the same rate when cultured with irradiated tumor cell lines expressing the Lym-1 and CD19 epitopes in the absence of exogenous cytokines (Figure 7).

The antitumor effects of Lym-1 CAR T cells were evaluated in a metastatic Raji/Luc-GFP xenograft model (Figure 8). A single dose of Lym-1 CAR T cells induced sustained tumor suppression in the NSG Raji xenograft mode, and a successful engraftment of human T cells in the NSG mice. In addition to ADCC and/or CDC effector mechanisms, chLym-1 is also able to induce cell apoptosis directly upon binding antigen [21]. This effect is caspase independent, and partially attributed to a rapid loss of mitochondrial membrane potential [21] and cell autophagy [22]. To determine if this effect is sufficient to eradicate the Raji tumor cells in this model, three doses of chLym-1 were administered. Although Raji tumor growth was initially checked, dramatic tumor progression was observed after day 20 (Figure 8). This limited suppression may arise because the half-life of the chimeric antibody is less than that of the CAR T cells that proliferate upon recognizing a target. Nonetheless, the apoptotic effect of Lym-1 binding to lymphoma cells may confer enhanced target killing on Lym-1 CAR T cells compared to CAR T cells based on an antibody without this effect.

The data presented here demonstrate that primary human T cells can be efficiently transduced with a Lym-1 CAR. In culture systems, Lym-1 CAR T cells recognize human lymphoma cell lines in an epitope-driven manner to secrete cytokines, proliferate, and kill tumor cells. Lym-1 CAR T cells eradicated Lym-1 epitope positive tumors in NSG mice. Lym-1 CAR T cells thus hold promise for the treatment of lymphomas in patients.

## 4. Materials and Methods

### 4.1. Antibodies

PE-anti-huCD107a (Clone: H4A3, Biolegend, San Diego, CA, USA), PE-anti-huCD3 (Clone: UCHT1, BD), APC-anti-huCD45 (Clone: H130, BD), Lym-1 and chLym-1 (lot: 100226) were produced in our laboratory [27], IL-7-Fc and IL-15-Fc were produced through a mammalian expression system and purified in-house.

### 4.2. Cell Lines

K562, Daudi, Jurkat, and Raji cell lines were obtained from American Type Culture Collection (ATCC, Manassas, VA, USA). Raji/Luc-GFP cells were a gift from Dr. Yvonne Y. Chen at the University of California, Los Angeles [28]. All cell lines were cultured in RPMI-1640 supplemented with 10% dialyzed fetal calf serum (FCS) (dFCS, Hyclone, Logan, UT, USA), 2% glutamine and 1% penicillin/streptomycin (Gemini Bio-Products, West Sacramento, CA, USA). HEK-293 LTV cells (Cell Biolabs Inc., San Diego, CA, USA) cultured in DMEM (Corning, Manassas, VA, USA) supplemented with 10% dFCS, 2% glutamine and 1% pen/strep were used for lentivirus production.

### 4.3. Vector Construction and Preparation of Lentivirus

For comparison purposes, a second generation CD19 CAR comprised of a CD19 specific targeting scFv derived from antibody FMC63, a CD8α leader sequence, a (G4S)3 linker between the VH and the VL domains, a CD8α hinge, a CD8α transmembrane domain followed by 4-1BB co-stimulatory domain, and intact intracellular CD3ζ, were cloned between the EcoRI and MluI restriction sites into the lentiviral vector pLVX-EF1α-IRES-Zsgreen (Clontech, Mountain View, CA, USA). For this study, the IRES-ZsGreen moiety was removed by restriction enzyme digestion with EcoRI and MluI (NEB, Ipswich, MA, USA), prior to insertion of the CAR encoding gene. The pLVX-EF1α-Lym-1 construct was made by substituting a scFv derived from Lym-1 for the FMC63 scFv in the CD19 vector (described above). Lentivirus was produced by transient co-transfection of the CAR transfer vectors (pLVX-EF1α-CD19 or pLVX-EF1α-Lym1) with packaging plasmids, psPAX2 and pMD2.G (Addgene) using HEK-293LTV cells. Supernatants containing viral particles were collected at 24 and 48 h after transfection, and were combined, filtered, and concentrated by ultracentrifugation. Pelleted virus was then resuspended in phosphate buffered saline (PBS) supplemented with 1% BSA and 7% trehalose, aliquoted, and stored at −80 °C. Viral titers were measured by transducing 10^6^ Jurkat T cells with 10-fold serial dilutions of virus vector. Forty-eight hours after transduction, cells were labeled with biotinylated Protein-L (Genscript, Piscatawat, NJ, USA) and detected by APC-labeled streptavidin (BD, Franklin Lakes, NJ, USA). Positively transduced cells at a range of 10–20% were used to calculate the virus transducing units (TU) by the following formula: TU/mL = (10^6^ seeded cells × % positive cells × 1000)/μL of virus vector.

### 4.4. Primary T Cell Isolation and Transduction

Blood from healthy donors was obtained using heparin collection tubes in accordance with IRB regulations at the University of Southern California (Protocol #11606, 8 January 2016). Primary blood mononuclear cells (PBMCs) were isolated using Ficoll-Paque (Life Technologies, Inc., Carlsbad, CA, USA) as per the manufacturer’s protocol. T cells were then isolated using a T cell negative selection kit (Stem Cell Technologies, Seattle, WA, USA) and cultured in T-cell medium (43% Clicks, 43% RPMI 1640, 2% Glutamax (Life Technologies, Inc., Carlsbad, CA, USA), 10% dFCS, 1% non-essential amino acid solution, 1% pen/strep solution, 50 ng/mL IL-7-Fc, 50 ng/mL IL-15-Fc). Three days prior to transduction, T cells were activated by adding CD3/CD28 beads (Life Technologies, Inc., Carlsbad, CA, USA) at a 1:1 ratio, and then transduced by centrifugation at 800 g for 90 min with lentivirus (MOI = 15) and LentiBlast (OZ bioscience, San Diego, CA, USA). Transduction was performed twice, followed by a media change after 24 h, after which cells were transferred to 24-well G-Rex plates (Wilson Wolf, St. Paul, MN, USA) supplemented with fresh T cell medium. T cells were used for assays at day 10 after transduction. CAR virus transduction efficiency was evaluated by flow cytometry at day 10 using biotinylated protein L followed by detection with APC-labeled streptavidin. Mock transductions were conducted as negative controls, as described above, but in the absence of a viable virus vector.

### 4.5. Cytotoxicity

Transduced effector CAR T-cell preparations were adjusted to be 30% positive for CAR T cells by addition of mock T cells. These adjusted preparations were incubated with target K562, Daudi, and Raji cells with ratios of CAR T cells to targets of 20:1, 10:1, 5:1, and 1:1 each, in flat-bottomed 96 well plates for 24 h, in the absence of added cytokines. Supernatants were collected and subjected to measurement of LDH activity (Pierce, Waltham, MA, USA), according to the manufacturer’s instructions.

### 4.6. Cytokine Production and Degranulation Assays

Target cells (10^5^) and 2 × 10^5^ CAR T cells (30% CAR positive) were co-cultured in 200 µL T-cell medium without addition of cytokines. After 24 h, supernatants were collected and analyzed for levels of INF-γ and IL-2 by ELISA (Life Technologies Corporation, Grand Island, NY, USA). For the CD107a degranulation assay, CAR T cells (30% CARs positive) and target cells were mixed at defined ratios, as described above. Five microliters of APC conjugated anti-huCD107a antibodies and 1× monensin (BioLegend, San Diego, CA, USA) were directly added to each well. After incubation for 5 h at 37 °C, cells were labeled with 5 µL PE-anti-huCD3 antibody, and subjected to flow cytometry using an Attune Acoustic Focusing Cytometer (Life Technologies, Carlsbad, CA, USA) to detect CD107a expression.

### 4.7. CFSE Proliferation Assay

T cells (10^6^) transduced either with the pLVX-EF1a-Lym-1 (30% CAR positive) or pLVX-EF1a-CD19 (30% CAR positive) were labeled with CFSE-Far-red, as per manufacturer’s instructions (Invitrogen, Carlsbad, CA, USA), and co-cultured with either irradiated K562, Raji, or Daudi cells at a ratio of 1:1 in 24 well G-Rex plates, in the absence of cytokines for 5 days. A flow cytometer was used to measure the cell proliferation profile. Irradiated cells were prepared by exposing 10^7^ cells to a total dose of 100 Gy using an X-RAD 320 (PXI, North Branford, CT, USA). After irradiation, cells were washed and cryopreserved by using a freezing solution (Cryostor CS10, Bothell, WA, USA), and stored at −80 °C.

### 4.8. Raji/Luc-GFP Xenograft Studies in NOD Scid-IL2Rgamma^null^ (NSG) Mice

All mouse experiments were approved by the USC Animal Care and Use Committee (Protocol #20585, 8 January2017). Eight-week-old male NSG mice purchased from Jackson Laboratories (JAX #3877458, 29 March 2016) were housed in the USC vivarium in sterile cages. Raji/Luc-GFP cells (10^6^) in 100 µL PBS were injected iv via the lateral tail vein using an insulin syringe (designated as day 0). Luciferase activity was measured on day 6 via bioluminescence imaging to assess tumor burden. On day 7, 10^7^ mock-transduced T cells, CD19 CAR T cells (50% CAR positive), or Lym-1 CAR T cells (50% CAR positive) were prepared in 100 µL PBS, and injected iv using an insulin syringe. A chimeric Lym-1 antibody control group was included, in which 100 μg chLym-1 in 100 µL PBS was injected iv on days 7, 9, and 11. Tumor progression was monitored by bioluminescence imaging using an IVIS imaging system at the USC Molecular Imaging Center. At day 60, surviving mice were euthanized, spleen and bone marrow cells harvested and re-suspended in a total volume of 2 mL of Flow cytometry (FACS) buffer (PBS, supplemented with 2% FCS). Two hundred microliters of the cell suspension were then labeled with PE-ani-huCD3 and APC-anti-huCD45 antibodies, and analyzed by flow cytometry to determine the percentage of human T cells.

### 4.9. Statistical Analysis

All results were expressed as means ± standard deviation (SD) or standard error of the mean (SEM), as indicated. Statistical analyses were performed using unpaired two-tailed Student’s *t*-test by GraphPad Prism 6.

## 5. Patents

Patents related to this work are published under publication numbers US 20160355590 (3 June 2016) and WO2016197064A1 (4 June 2015) entitled: Lym-1 and Lym-2 Targeted CAR T Cell Immunotherapy.

## Abbreviations

ALL	acute lymphoblastic leukemia
ASCT	allogenic stem cell transplantation
B-NHL	B-cell non-Hodgkin lymphomas
CAR	chimeric antigen receptor
CLL	chronic lymphocytic leukemia
ORR	objective response rates
OS	overall survival
R/R	relapsed or Refractory
NHL	non-Hodgkin lymphoma
NSG	NOD scid IL2Rgamma^null^
